# Prognostic Role of Metabolic Syndrome in COVID-19 Patients: A Systematic Review Meta-Analysis

**DOI:** 10.3390/v13101938

**Published:** 2021-09-27

**Authors:** Marco Zuin, Gianluca Rigatelli, Claudio Bilato, Carlo Cervellati, Giovanni Zuliani, Loris Roncon

**Affiliations:** 1 Department of Translational Medicine, Faculty of Medicine, University of Ferrara, 44100 Ferrara, Italy; marco.zuin@edu.unife.it (M.Z.); claudio.cervellati@unife.it (C.C.); giovanni.zuliani@unife.it (G.Z.); 2 Department of Cardiology, Santa Maria Della Misericordia Hospital, 45100 Rovigo, Italy; jackyheart@libero.it; 3 Division of Cardiology, West Vicenza General Hospitals, Arzignano, 36071 Vicenza, Italy; claudio.bilato@aulss8.veneto.it

**Keywords:** metabolic syndrome, COVID-19, prevalence, mortality, dyslipidaemia

## Abstract

Background: The prevalence and prognostic implications of metabolic syndrome (MetS) in patients infected by the SARS-CoV-2 remain unclear. We performed a systematic review and meta-analysis of prevalence and mortality risk in COVID-19 patients with MetS. Methods: Preferred Reporting Items for Systematic Reviews and Meta-Analyses guidelines were followed in abstracting data and assessing validity. We searched MEDLINE and Scopus to locate every article published up to 1 September 2021, reporting data on MetS among COVID-19 patients. The pooled prevalence of MetS was calculated using a random effects model and presented using the related 95% confidence interval (CI), while the mortality risk was estimated using the Mantel-Haenszel random effects models with odds ratio (OR) and related 95% CI. Statistical heterogeneity was measured using the Higgins I^2^ statistic. Results: Six studies, enrolling 209.569 COVID-19 patients [mean age 57.2 years, 114.188 males (54.4%)] met the inclusion criteria and were included in the final analysis. The pooled prevalence of dyslipidaemia was 20.5% of cases (95% CI: 6.7–47.8%, *p* = 0.03), with high heterogeneity (I^2^ = 98.9%). Pre-existing MetS was significantly associated with higher risk of short-term mortality (OR: 2.30, 95% CI: 1.52–3.45, *p* < 0.001), with high heterogeneity (I^2^ = 89.4%). Meta-regression showed a direct correlation with male gender (*p* = 0.03), hypertension (*p* < 0.001), DM (*p* = 0.01) and hyperlipidaemia (*p* = 0.04), but no effect when considering age (*p* = 0.75) and chronic pulmonary disease (*p* = 0.86) as moderators. Conclusions: MetS represents a major comorbidity in about 20% of COVID-19 patients and it is associated with a 230% increased risk of short-term mortality.

## 1. Introduction

Since the beginning of COVID-19 outbreak, a growing body of evidence has demonstrated that clinical outcomes in patients with SARS-CoV-2 infection are closely related to the burden of associated comorbidities [1], such as arterial hypertension (HT), diabetes mellitus (DM) and cardiovascular disease (CVDs) [2,3,4,5]. Notably, most of these concomitant diseases constitute the definition of metabolic syndrome (MetS), a frequent metabolic disorder in the general population, which is commonly considered as a risk factor for the progression of CVDs and type 2 diabetes [6]. Although some recent analyses have investigated the role of MetS in COVID-19 patients, a comprehensive assessment of data regarding the real prevalence and prognostic role of MetS in SARS-CoV-2 infected individuals has not yet been performed. The aim of the present study is to estimate the pooled prevalence and the influence of MetS on short-term mortality in COVID-19 patients by a systematic review and meta-analysis of the available data.

## 2. Methods

### 2.1. Data Sources and Searches

The study was performed in accordance with the Preferred Report Items for Systematic Reviews and Meta-analyses (PRISMA) guidelines (Supplementary File S1) [7]. PubMed and Scopus databases were systematically searched for articles, published in the English language, from inception through 1 September 2021 with the following Medical Subject Heading (MESH) terms: “COVID-19” OR “SARS-CoV-2” AND “Metabolic Syndrome”, following the same PICO (population, intervention, comparison, and outcome) strategy (Table 1).

In addition, references from the included studies were screened to potentially identify other investigations meeting the inclusion criteria.

### 2.2. Study Selection

Specifically, inclusion criteria were: (i) studies enrolling subjects with a confirmed diagnosis of COVID-19; (ii) studies providing data on the prevalence of MetS among patients enrolled and (iii) adjusted odds ratios (aORs) estimating the short-term mortality risk among COVID-19 patients with MetS. Conversely, case reports, review articles, abstracts, editorials/letters and case series with less than 10 participants were excluded. Each included article was independently evaluated by two reviewers (MZ, LR); in case of discrepancies a third author was involved (CB), and final consensus was achieved through discussion.

### 2.3. Data Extraction and Quality Assessment

Data were independently extracted by two reviewers (MZ, GR) using a standardized protocol. Disagreements were resolved. For this meta-analysis, the following data elements were extracted: sample size, mean age, number of non-survivors (NS), male gender, prevalence of MetS, major comorbidities such as HT, DM, obesity, chronic pulmonary disease; Hyperlipidaemia and aOR for short-term mortality in COVID-19 patients with MetS. The quality of included studies was graded using the Newcastle–Ottawa quality assessment scale (NOS) [8].

### 2.4. Outcomes

The prevalence of MetS in COVID-19 patients was chosen as the primary outcome while its associated mortality risk was selected as the secondary outcome.

### 2.5. Data Synthesis and Analysis

Continuous variables were expressed as mean or as a median while categorical variables as counts and percentages. The cumulative prevalence of MetS (*n*/*N*), defined as the ratio between patients with pre-existing Mets (n) and the number of patients enrolled in each study (N), were pooled using a random effects model and presented with the corresponding 95% confidence interval (CI). To estimate the mortality risk, data were pooled using the Mantel-Haenszel random effects models with odds ratio (OR) as the effect measure with 95% CI. Heterogeneity among studies was assessed using Higgins and Thomson I^2^ statistic where I^2^ values corresponding with the following levels of heterogeneity: low (<25%), moderate (25–75%) and high (>75%) [9]. The presence of potential publication bias was verified by visual inspection of the funnel plot. Due to the low number of included studies (<10), small-study bias was not examined as our analysis was underpowered to detect such bias. A predefined sensitivity analysis (leave-one-out analysis) was performed removing one study at the time to evaluate the stability of our results regarding the mortality risk. To further appraise the impact of potential baseline confounders, a meta-regression analysis using age, gender, HT, DM, chronic pulmonary disease and hyperlipidaemia as moderator variables was performed. These variables were selected since previous analyses have identified those comorbidities as independent predictors of mortality in COVID-19 patients [2,3,10,11]. All meta-analyses were conducted using Comprehensive Meta-Analysis software, version 3 (Biostat, Englewood, NJ, USA).

## 3. Results

### 3.1. Search Results

**Figure 1:**
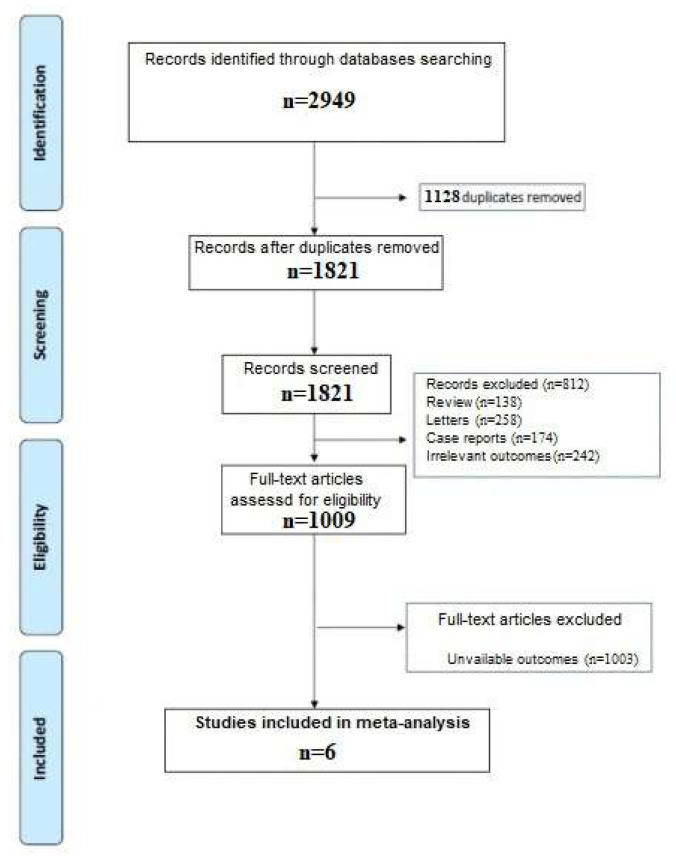
PRISMA flow diagram.

A total of 2949 articles were obtained by our search strategy. After excluding duplicates and preliminary screening, 1009 full-text articles were assessed for eligibility and 812 studies were excluded for not meeting the inclusion criteria while 1003 for unavailable outcomes, leaving six investigations fulfilling the inclusion criteria [12,13,14,15,16,17]. A flow diagram of the literature search and related screening process is shown in Figure 1.

### 3.2. Study Characteristics

Overall, 209,569 COVID-19 patients (mean age 57.2 years, 114,188 males (54.4%)) were included in the analysis. The general characteristics of the included studies are summarized in Table 2. Despite the concomitant comorbidities were not systematically investigated by the studies revised, HT and DM were the most frequently observed *n* = 43,987 (20.9%) and *n* = 35,124 (16.7%) for HT and DM, respectively. Quality assessment showed that all the studies were of moderate-high quality according to the NOS scale. The clinical characteristics considered by each revised study to define the presence of MetS are presented in Table 3.

### 3.3. Pooled Prevalence of Metabolic Syndrome

**Figure 2:**
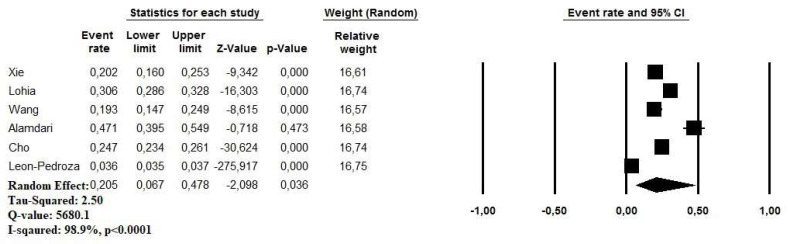
Pooled prevalence of pre-existing metabolic syndrome in COVID-19 patient.

**Figure 3:**
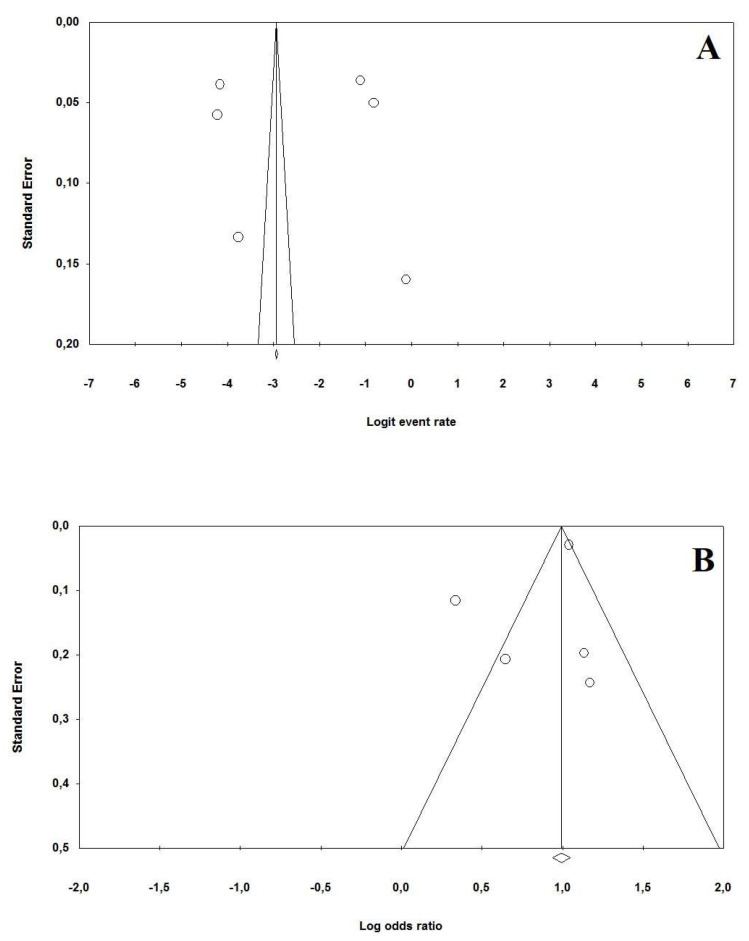
Funnel plots for (**A**) the polled prevalence of metabolic syndrome in COVID-19 patients and (**B**) for the mortality risk due to metabolic syndrome in COVID-19 patients.

The prevalence of MetS among COVID-19 patients ranged between 3.6% and 47.1%. A random effect model revealed a pooled prevalence of MetS in 20.5% of cases (95% CI: 6.7–47.8%, *p* = 0.03). A high heterogeneity was observed in the analysis (I^2^ = 98.9%) (Figure 2). The relative funnel plot is presented in Figure 3.

### 3.4. Dyslipidaemia and Mortality Risk

Five studies reported an aOR for the mortality risk in COVID-19 patients with Mets (*n* = 209,336 patients, mean age 59.2 years, 113,786 males) [12,13,15,16,17]. The variables used by each study to determine the aOR for the short-term mortality risk are presented in Table 4. On pooled analysis, patients with MetS showed a significantly higher mortality risk in the short-term period (OR: 2.30, 95% CI: 1.52–3.45, *p* < 0.001, I^2^ = 89.4%) (Figure 3). The relative funnel plot is presented in Figure 3B.

### 3.5. Sensitivity Analysis and Publication Bias

**Figure 4:**
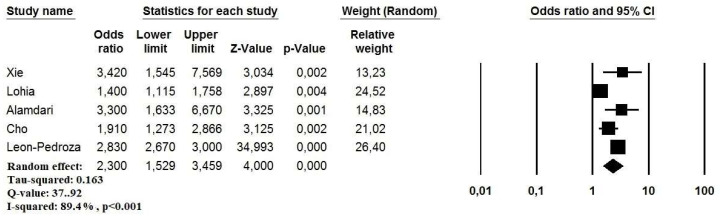
Forest plot investigating the mortality risk due to metabolic syndrome in COVID-19 patients using a random-effect model.

To evaluate the robustness of the association results, we performed a leave-one-out sensitivity analysis by iteratively removing one study at a time and recalculating the summary OR. The summary ORs remained stable (ranging between OR: 2.24, 95%CI: 1.12–2.88, *p* = 0.001 and OR: 2.35, 95%CI: 1.62–3.48, *p* < 0.001), indicating that our results were not driven by any single study. Due to the limited number of studies available to perform the meta-analysis, funnel plots cannot reassure the presence of potential publication bias, since the power of the test was too low in distinguishing chance from real asymmetry (Figure 4).

### 3.6. Meta-Regression

Meta-regression analysis revealed a direct relationship between the risk of short-term mortality and MetS in COVID-19 patients using male gender (*p* = 0.03), HT (*p* < 0.001), DM (*p* = 0.01) and hyperlipidaemia (*p* = 0.04). Conversely, there was no association when age (*p* = 0.75) and the prevalence of chronic pulmonary disease was considered as moderator (*p* = 0.86).

## 4. Discussion

Three major findings emerge from the present meta-analysis based on more 200,000 patients. Firstly, Mets is present in about one out of four COVID-19 patients. Secondly, and more importantly, SARS-CoV-2 infected patients with a pre-existing MetS had an approximately 230% higher risk of death in the short-term period compared to those without. Thirdly, this last association was directly influenced by male gender, HT, DM and hyperlipidaemia.

Our results are in accordance with previous investigations examining the prognostic role of single MetS components, such as HT, DM, dyslipidaemia and obesity [2,3,5,21], reinforcing the concept that cardio-metabolic comorbidities play a pivotal role in determining the COVID-19 patient’s outcome. Unfortunately, the revised studies did not systematically report the aOR regarding the mortality risk for each MetS constituent, therefore we were not able to assess the relative single contribution in determining the global risk. However, this later aspect is partially mitigated by the large number of results derived by recent studies linking the single MetS components with a poor outcome in COVID-19 patients [2,3,5,21]. Performing a meta-regression based on such a disease constituting the definition of MetS, it emerged that HT and DM were those that significantly increased the mortality risk, as observed by Leon-Pedroza et al. [17]. Intriguingly, two of the revised studies also demonstrated that the mortality risk increased according to the number of MetS components [15,17]. On the other hand, both age and male gander have been identified as independent predictors of worse outcomes in patients with SARS-CoV-2 infection [22,23]. The high heterogeneity observed in our study is probably multifactorial. To this regard, the limited number of studies satisfying the inclusion criteria and the relative few numbers of enrolled patients represent, per se, a potential source of heterogeneity. Secondly, inherited biases derived from the original investigations may have further contributed to the heterogeneity level observed (methodological heterogeneity). In fact, different levels of methodological quality, sampling methods and definitions of MetS may have produced significant difference among the studies. Although some part of the heterogeneity between studies could be explained by the results of the meta-regression performed, our findings must be considered as preliminary, needing further confirmation from larger and randomized studies. What is certain is that, from a pathophysiological perspective, both aging and chronic metabolic disease, such as MetS, dysregulates the immune function leading to a chronic inflammatory state predisposing a perfect cytokine storm during COVID-19 infection [24] which influences the outcome of these patients [25].

Notably, some of the components of MetS are also related to low vitamin D level. In fact, a significant association between vitamin D deficiency and MetS has already been described in the general population [26]. To this regard, vitamin D deficiency seems to be common among COVID-19 patients, playing a significant role in worsening the prognosis of these patients [27,28,29]. However, none of the studies reviewed presented data on vitamin D status. Therefore, our results confirm that the metabolic status assessment remains important in the prognosis of these patients and in the risk of infection [30]. Our findings may have important implications in daily clinical practice, indeed, the prompt identification of patients with pre-existing MetS remains critical to promptly identify vulnerable populations who would require prioritization in treatment and prevention, and close monitoring if infected.

However, the presented results must be considered as preliminary, due the limited number of studies available on the relationship between MetS and COVID-19 mortality. Further investigations on this issue are urgently necessary to confirm our results.

## 5. Limitations

Our study has several limitations related to the design of the studied reviewed with all inherited biases and the numbers of investigation on the issue. In fact, only a few studies have analysed the relationship between MetS and mortality in COVID-19 patients, limiting our results and conclusions. Moreover, the relatively high heterogeneity observed, which probably depends on the inclusion criteria, as well as by the studies’ design and different criteria used to define the presence of MetS, may have resulted in the absence of firm conclusions. Furthermore, the potential underestimation of pre-existing MetS in hospitalized patients may have distorted our results. Further larger clinical studies are needed to confirm our preliminary results, as well as to compare the prognostic impact of individual comorbidities that constitute the definition criteria of MetS.

## 6. Conclusions

MetS is present in about 20% of patients with COVID-19 infection and is associated with an increased risk of short-term mortality Present results further reinforce the concept that cardiovascular comorbidities and risk factors play a pivotal role in determining the COVID-19 patient’s outcome [5]. However, this finding must be cautiously interpreted because of the high heterogeneity observed.

## Figures and Tables

**Table 1:** PICO criteria description and research question defined to systematic review.

Parameter	Description
Popualation	COVID-19 patients
Indicator	Presence of MetS
Comparison	COVID-19 patients with vs without MetS
Outcome	Short-term mortality
Research question	Is MetS associated with higher mortality in COVID-19 patients?

**Table 2:** General characteristics of the population enrolled. NS: Non-survivors; MetS: Metabolic syndrome; HT: Arterial hypertension; DM: Diabetes Mellitus; CPD: Chronic obstructive pulmonary disease; NOS: Newcastle-Ottawa quality assessment scale.

Author	N° of Pts	Age (Years)	Males, n (%)	NS, n (%)	MetS, n (%)	HT, n (%)	DM, n (%)	Obesity, n (%)	CPD, n (%)	Hyperlipidaemia, n (%)	NOS
Xie et al. [12]	287	61.5	124 (43.2)	58 (20.1)	188 (66.0)	230 (80.1)	154 (53.6)	187 (65.2)	29 (10.1)	122 (39.0)	8
Lohia et al. [13]	1.871	66.0	965 (51.6)	613 (32.8)	573 (30.6)	1485 (79.4)	792 (42.3)	879 (47.0)	317 (16.9)	513 (27.4)	8
Wang et al. [14]	233	47.0	132 (56.7)	0	45 (19.3)	38 (16.3)	18 (7.7)	NR	10 (4.3)	NR	7
Alamdari et al. [15]	157	68	138 (87.8)	NR	74 (47.1)	97 (61.7)	78 (49.6)	NR	42 (26.7)	51 (32.4)	7
Cho et al. [16]	4.070	55.9	1530 (37.6)	142 (3.4)	1007 (24.7)	1323 (32.5)	590 (14.5)	NR	NR	1386 (34.1)	7
Leon-Pedroza et al. [17]	202.951	45	111.299 (54.8)	25.060 (12.3)	7308 (3.6)	40.814 (20.2)	33.492 (16.6)	39.873 (19.7)	3.607 (1.8)	NR	8

**Table 3:** Criteria used by the revised studies to define the metabolic syndrome. BMI. Body mass index; TG: Triglycerides; HDL: High-density lipoprotein.

Author	Criteria Used to Define MetS	N° of Criteria to Define MetS
Xie et al. [12]	Modified WHO criteria [6]	At least three
Lohia et al. [13]	History of diabetes or use of antidiabetics; Obesity (BMI > 30); History of hypertension or use of antihypertensive medications; TG > 150 mg/dL; HDL < 40 in males and <50 in females History of hypercholesterolemia and use of cholesterol-lowering drugs	
Wang et al. [14]	Modified WHO criteria [6]	At least three
Alamdari et al. [15]	National Cholesterol Education Program (NCEP)—Adult Treatment Panel (ATPIII) criteria [18]	At least three
Cho et al. [16]	American guidelines iof the Inernational Diabetes Federation and American Heart Association [19]	At least three
Leon-Pedroza et al. [17]	Sperling et al. [20]	At least three

**Table 4:** Variables used in the revised studies to calculate the adjusted odd ratio for the mortality risk in COVID-19 patients with metabolic syndrome. CAD: Coronary artery disease; COPD: Chronic obstructive pulmonary disease; CKD: Chronic kidney disease; ESRD: End-stage renal disease; HT: Arterial hypertension; HDL: High-density lipoprotein.

Author	Adjustement Variables
Xie et al. [12]	Age, gender, race, hospital site, Charlson Comorbidity index
Lohia et al. [13]	Age, gender, race, smoking, insurance, CAD, COPD, asthma, CKD, ESRD, cancer, liver disease, previous stroke
Wang et al. [14]	Not evaluated for the secondary outcome of the study since no death were registered in the original study
Alamdari et al. [15]	Wrist circumference, increased blood glucose, HT, low HDL, elevated triglycerides
Cho et al. [16]	Age, gender, region, social economic status, smoking, alcohol, physical activity, cardiovascular disease, asthma, atrial fibrillation, chronic kidney disease, cancer and non-alcoholic fatty liver disease
Leon-Pedroza et al. [17]	Age, sex

## Data Availability

All the data pertaining to the study are available in the manuscript.

## Supplementary Materials

The following are available online at https://www.mdpi.com/article/10.3390/v13101938/s1. Table S1. PRISMA Checklist.
